# A 24-hour landmark machine learning model for predicting new-onset sepsis-associated encephalopathy

**DOI:** 10.3389/fmed.2026.1904524

**Published:** 2026-08-11

**Authors:** Weiwei Hong, Ziming Jiang, Jiahui Ruan, Danfeng Weng, Ruijuan Zhang, Liang Dong

**Affiliations:** 1 Taizhou Central Hospital (Taizhou University Hospital), Taizhou, Zhejiang Province, China; 2 Open College, Taizhou Open University, Taizhou, Zhejiang Province, China

**Keywords:** intensive care unit, logistic regression, machine learning, sepsis, sepsis-associated encephalopathy

## Abstract

**Background:**

Sepsis-associated encephalopathy (SAE) is a common neurological complication of sepsis and is associated with prolonged intensive care unit (ICU) stay, extended mechanical ventilation, and poor outcomes. This study aimed to develop and internally validate a 24-hour landmark machine learning-based model for predicting new-onset SAE after the first ICU day in patients with sepsis.

**Methods:**

This retrospective observational study included 537 ICU patients meeting the Sepsis-3 criteria. This retrospective study developed a 24-hour landmark prediction model. Clinical variables collected during the first 24 h after ICU admission were used to predict new-onset SAE occurring after the 24-hour landmark time point among patients who survived, remained in the ICU, and were SAE-free during the first ICU day. Demographic characteristics, comorbidities, vital signs, laboratory parameters, organ function scores, therapeutic interventions, and prognosis-related variables were collected. Missing data were handled by multiple imputation. The cohort was randomly divided into a training set and an internal test set at a 7:3 ratio. Model performance was evaluated using internal validation only. Six models were developed, including logistic regression, random forest, XGBoost, support vector machine, k-nearest neighbors, and naive Bayes. Model performance was assessed using discrimination, calibration, and decision curve analysis.

**Results:**

SAE occurred in 215 patients, corresponding to an incidence of 40.0%. Compared with non-SAE patients, patients with SAE had greater disease severity, higher SOFA and APACHE II scores, increased respiratory rate, worse renal and inflammatory profiles, and higher rates of vasoactive drug use, mechanical ventilation, and continuous renal replacement therapy. In the internal test set, logistic regression showed the highest AUC among the candidate models, with an AUC of 0.767, accuracy of 0.698, and sensitivity of 0.754. Calibration analysis showed acceptable agreement between predicted and observed risks, and decision curve analysis indicated potential net benefit. A nomogram was constructed to visualize the final model.

**Conclusion:**

The logistic regression-based model showed moderate predictive performance and may support preliminary 24-hour landmark risk stratification for new-onset SAE in ICU patients with sepsis. Further multicenter prospective validation is required.

## Introduction

Sepsis is a life-threatening syndrome characterized by organ dysfunction caused by a dysregulated host response to infection (1). Despite advances in critical care, sepsis remains a major global health burden. A Global Burden of Disease analysis estimated that 48.9 million incident cases of sepsis and 11.0 million sepsis-related deaths occurred worldwide in 2017, accounting for nearly one fifth of all global deaths (2). Early identification of organ dysfunction and timely implementation of evidence-based management are therefore central priorities in the care of patients with sepsis (3).

Sepsis-associated encephalopathy (SAE) is one of the most common and clinically important neurological complications of sepsis. It is generally defined as diffuse cerebral dysfunction secondary to sepsis in the absence of direct central nervous system infection, structural brain disease, or other identifiable causes of encephalopathy (4, 5). The clinical spectrum of SAE ranges from subtle inattention and delirium to stupor and coma, and its assessment in the intensive care unit (ICU) is often complicated by sedation, mechanical ventilation, metabolic disturbances, and pre-existing neurological conditions (5, 6). Previous studies have reported that SAE may occur in approximately 30% to 70% of patients with sepsis, depending on disease severity, population characteristics, and diagnostic criteria (5, 7). Importantly, SAE is not merely a transient neurological manifestation; it has been associated with increased short-term mortality, prolonged ICU stay, higher medical resource utilization, and long-term cognitive impairment among survivors (4, 7, 8).

The pathophysiology of SAE is complex and multifactorial. Proposed mechanisms include systemic and neuroinflammation, blood-brain barrier disruption, endothelial dysfunction, microcirculatory impairment, neurotransmitter imbalance, mitochondrial dysfunction, metabolic derangements, and neuronal apoptosis (4, 6, 9). However, no specific treatment for SAE has been established, and current management mainly relies on early recognition, treatment of the underlying infection, optimization of organ perfusion and oxygenation, correction of metabolic abnormalities, and prevention or management of delirium (5, 6). Therefore, identifying patients with sepsis who are at high risk of developing SAE after the first ICU day may help clinicians intensify neurological monitoring, optimize modifiable risk factors, and improve individualized decision-making.

Traditional clinical prediction approaches may be limited in SAE because of the heterogeneous presentation of sepsis, non-linear interactions among physiological variables, and the dynamic nature of ICU data. Machine learning methods can integrate high-dimensional clinical information and capture complex relationships among demographic characteristics, comorbidities, vital signs, laboratory results, organ support, and severity scores. Recent studies have explored machine learning models for predicting SAE occurrence or outcomes in critically ill patients, suggesting that routinely collected electronic health record data may support early risk stratification (10, 11). Nevertheless, further model development and validation are needed, particularly with attention to discrimination, calibration, potential net benefit, and interpretability, before such tools can be considered for clinical implementation.

Therefore, this study aimed to develop and validate a machine learning-based model for predicting SAE in patients with sepsis. By using routinely available clinical variables, we sought to construct an accurate and interpretable prediction model that may support preliminary risk stratification of patients at risk for new-onset SAE after the first ICU day and provide a basis for timely preventive and therapeutic strategies.

## Materials and methods

### Data source

This study was conducted and reported in accordance with the transparent reporting of a multivariable prediction model for individual prognosis or diagnosis (TRIPOD) statement (12, 13). We retrospectively collected data from patients diagnosed with sepsis who were admitted to the ICU of Taizhou Central Hospital between January 2023 and December 2025. The study protocol was approved by the Ethics Committee of Taizhou Central Hospital (approval number: 2026L-01-07).

### Study population

**Figure 1:**
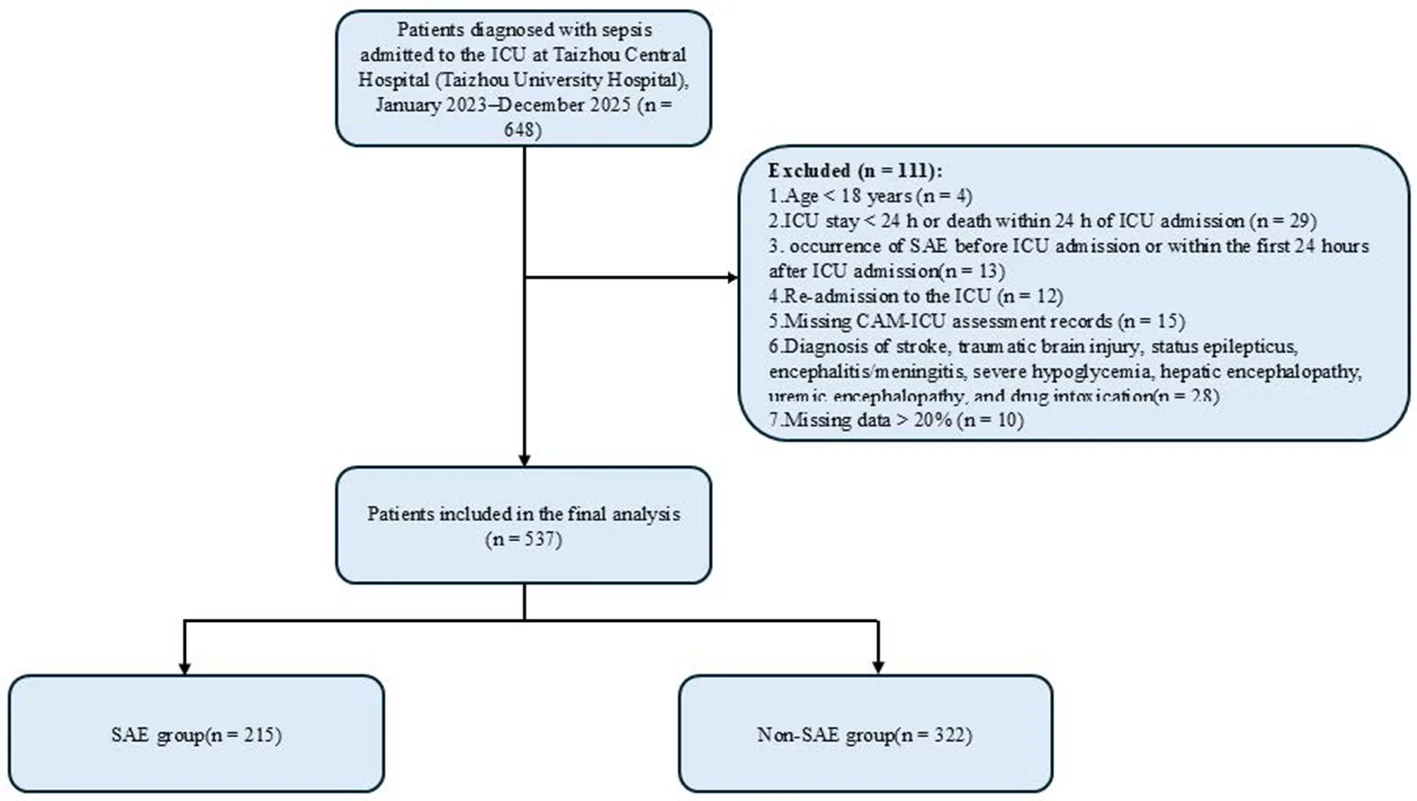
Flowchart of the study.

Adult ICU patients meeting the Sepsis-3 criteria were eligible for inclusion. Sepsis was defined as suspected or confirmed infection accompanied by acute organ dysfunction, indicated by an increase in the Sequential Organ Failure Assessment (SOFA) score of ≥2 points (1). In this study, SAE was operationally defined as acute brain dysfunction occurring after the first 24 h of ICU admission in patients with sepsis, identified by GCS < 15 and positive CAM-ICU assessment, after excluding direct central nervous system infection, structural brain disease, severe hypoglycemia, hepatic encephalopathy, uremic encephalopathy, and drug intoxication. CAM-ICU assessments were performed every 8 h by professionally trained nursing staff. Exclusion criteria were as follows: 1. age < 18 years; 2. ICU length of stay < 24 h due to discharge or death; 3. patients with SAE diagnosed before ICU admission or during the first 24 h after ICU admission were excluded; 4. multiple ICU admissions during the same hospitalization, in which case only the first ICU admission was retained (or subsequent admissions were excluded); 5. absence of CAM-ICU assessment records; 6. diagnosis of stroke, traumatic brain injury, status epilepticus, encephalitis/meningitis, severe hypoglycemia, hepatic encephalopathy, uremic encephalopathy, and drug intoxication; 7. missing data exceeding 20% (Figure 1).

### Outcome definition

The outcome was new-onset SAE occurring after the first 24 h of ICU admission. SAE events identified before ICU admission or during the first 24 h after ICU admission were not considered prediction outcomes and were excluded from the landmark cohort.

### Data collection

Data for this study were obtained from the hospital information system (HIS). Predictor variables were extracted from the first 24 h after ICU admission, before the landmark time point. These variables were used to predict new-onset SAE after the first ICU day:

(1) Demographic variables: age, gender, and body mass index (BMI). (2) Comorbidities: hypertension, diabetes mellitus, myocardial infarction (MI), chronic heart failure (CHF), chronic pulmonary disease (CPD), liver disease, renal disease, malignancy tumor (MT), AIDS, and history of alcohol abuse. (3) Illness severity scores: SOFA score, Acute Physiology and Chronic Health Evaluation II (APACHE II) score, and Glasgow Coma Scale (GCS) score. (4) Vital signs and bedside monitoring variables: body temperature, heart rate (HR), respiratory rate (RR), systolic blood pressure (SBP), mean arterial pressure (MAP), diastolic blood pressure (DBP), peripheral oxygen saturation (SpO_2_), and blood glucose. (5) Laboratory variables: arterial blood gas and acid–base parameters (pH, PaCO_2_, PaO_2_, and lactate), complete blood count (white blood cell count, hemoglobin, and platelet count), coagulation parameters (INR and PT), electrolytes (serum potassium and sodium), renal function markers (blood urea nitrogen and creatinine), liver function markers (total bilirubin and albumin), inflammatory marker (C-reactive protein). (6) Therapeutic interventions within the first 24 h of ICU admission: use of mechanical ventilation, continuous renal replacement therapy (CRRT), and vasopressor agents. For continuous variables, mean values within the first 24 h after ICU admission were used for analysis.

### Statistical analysis

Continuous variables with a normal distribution are presented as mean ± standard deviation (SD) and were compared between groups using the independent-samples *t* test. Continuous variables with a non-normal distribution are reported as median (25th−75th percentile) and were compared using the Wilcoxon rank-sum test. Categorical variables are expressed as counts and percentages and were compared using the chi-square test. The cohort was randomly divided into a training set and an internal test set at a 7:3 ratio using stratified sampling before model development. Variables with < 20% missing data (Supplementary Figure S4) were imputed using multiple imputation by chained equations (14), within the training data only. We generated 20 imputed datasets with 20 iterations, using predictive mean matching for continuous variables, logistic regression for binary variables, and multinomial logistic regression for categorical variables. The imputation models and preprocessing parameters estimated from the training data were then applied to the internal test set. Imputation, preprocessing, scaling, feature selection, hyperparameter tuning, model fitting, and threshold selection were performed within the training data only. The internal test set was kept fully independent and was used only for final model evaluation. The primary performance metric was the area under the receiver operating characteristic curve (AUROC). The 95% confidence interval (CI) for the AUROC was estimated using bootstrap resampling with 1,000 iterations. Receiver operating characteristic (ROC) curves were generated, and accuracy, sensitivity, specificity, and F1 score were calculated to assess model discrimination. Model calibration was evaluated using calibration curves and the Brier score. Decision curve analysis (DCA) was performed to quantify the net clinical benefit of the models across a range of threshold probabilities. All analyses were performed using R Statistical Software (version 4.4.2) and Python (version 3.9).

## Results

### Baseline characteristics

A total of 537 patients with sepsis were included in the analysis, including 322 patients in the non-SAE group and 215 patients in the SAE group. The incidence of SAE was 40.0%. As shown in Table 1, there were no significant differences between the two groups in age, sex, BMI, alcohol use, hypertension, diabetes, congestive heart failure, chronic pulmonary disease, myocardial infarction, liver disease, renal disease, or malignant tumor (*P* > 0.05).

**Table 1:** Baseline characteristics.

Characteristics	Non-SAE (*N* = 322)	SAE (*N* = 215)	*P*-value
Age (years)	64.00 [52.00, 72.00]	65.00 [53.50, 74.00]	0.347
Male (%)	188 (58.39)	129 (60.00)	0.709
BMI (kg/m^2^)	29.77 [26.61, 33.30]	30.11 [26.85, 35.15]	0.104
Alcohol abuse (%)	36 (11.18)	14 (6.51)	0.068
Hypertension (%)	68 (21.12)	43 (20.00)	0.754
Diabetes (%)	115 (35.71)	77 (35.81)	0.981
CHF (%)	119 (36.96)	79 (36.74)	0.960
CPD (%)	72 (22.36)	61 (28.37)	0.114
MI (%)	49 (15.22)	42 (19.53)	0.191
Liver disease (%)	53 (16.46)	48 (22.33)	0.088
Renal disease (%)	91 (28.26)	71 (33.02)	0.239
Malignant tumor (%)	33 (10.25)	21 (9.77)	0.856
AIDS (%)	0 (0.00)	4 (1.86)	0.052
APACHE II	23.00 [18.00, 30.75]	30.00 [23.00, 37.00]	< 0.001
Modified APACHE II	21.00 [14.00, 28.00]	27.00 [20.00, 34.00]	< 0.001
SOFA	3.00 [2.00, 5.00]	4.00 [2.00, 5.00]	0.005
GCS	15.00 [14.00, 15.00]	14.00 [12.00, 14.00]	< 0.001
Temperature (°C)	36.86 [36.64, 37.16]	36.96 [36.66, 37.37]	0.035
RR (/min)	18.86 [16.69, 22.14]	22.06 [18.53, 25.03]	< 0.001
SpO_2_ (%)	97.22 [95.79, 98.66]	97.17 [95.61, 98.62]	0.504
SBP (mmHg)	111.79 [104.06, 121.57]	111.53 [102.67, 120.41]	0.483
DBP (mmHg)	59.95 [54.78, 66.58]	59.78 [54.29, 64.69]	0.518
MBP (mmHg)	99.50 [90.00, 113.75]	103.00 [90.00, 120.00]	0.217
HR (/min)	85.79 [77.40, 97.57]	89.62 [75.29, 100.25]	0.467
Glucose (mg/dl)	149.00 [131.32, 167.88]	151.40 [132.10, 172.80]	0.487
Wbc ( × 10^9^/L)	12.88 [9.35, 17.78]	12.80 [8.75, 19.45]	0.966
Hemoglobin (g/dl)	9.60 [8.40, 11.10]	10.05 [8.40, 11.65]	0.132
Platelet ( × 10^9^/L)	202.00 [139.62, 298.62]	197.00 [128.75, 287.00]	0.335
pH	7.37 [7.33, 7.42]	7.35 [7.29, 7.40]	0.001
PaO_2_ (mmHg)	148.25 [93.12, 226.88]	124.50 [95.75, 178.50]	0.024
PaCO_2_ (mmHg)	40.00 [35.50, 45.00]	40.00 [34.00, 46.00]	0.569
Lactate (mmol/L)	1.60 [1.20, 2.35]	1.70 [1.17, 2.60]	0.254
K (mmol/L)	4.20 [3.90, 4.69]	4.30 [3.90, 4.90]	0.249
Na (mmol/L)	137.00 [134.00, 139.00]	138.00 [133.50, 141.00]	0.036
PT (s)	15.75 [13.45, 18.64]	15.50 [13.45, 19.98]	0.542
INR	1.45 [1.25, 1.70]	1.40 [1.25, 1.85]	0.594
Alb (g/dl)	2.97 [2.70, 3.26]	2.98 [2.60, 3.30]	0.959
Cr (mg/dl)	1.15 [0.80, 1.89]	1.40 [0.90, 2.58]	0.008
Bun (mg/dl)	20.50 [14.00, 35.38]	27.00 [17.00, 49.75]	< 0.001
Tbil (mg/dl)	0.70 [0.45, 1.20]	0.70 [0.40, 1.42]	0.629
CRP (mg/l)	94.50 [35.65, 169.95]	120.90 [58.55, 213.90]	0.004
Vasopressor use (%)	143 (44.41)	121 (56.28)	0.007
Mechanical ventilation (%)	153 (47.52)	167 (77.67)	< 0.001
CRRT (%)	6 (1.86)	15 (6.98)	0.003

BMI, body mass index; CHF, chronic heart failure; CPD, chronic pulmonary disease; MI, myocardial infarction; GCS, Glasgow Coma Scale; SBP, systolic blood pressure; MAP, mean arterial pressure; DBP, diastolic blood pressure; HR, heart rate; RR, respiratory rate; INR, international normalized ratio; PT, prothrombin time; Modified APACHE II, modified APACHE II score without GCS.

Compared with patients without SAE, patients with SAE had significantly higher disease severity scores, including APACHE II score and SOFA score. Regarding vital signs and laboratory findings, patients with SAE had higher respiratory rate [22.06 (18.53, 25.03) vs. 18.86 (16.69, 22.14) breaths/min, *P* < 0.001], slightly higher temperature [36.96 (36.66, 37.37) vs. 36.86 (36.64, 37.16) °C, *P* = 0.035], lower pH [7.35 (7.29, 7.40) vs. 7.37 (7.33, 7.42), *P* = 0.001], and lower PaO_2_ [124.50 (95.75, 178.50) vs. 148.25 (93.12, 226.88) mmHg, *P* = 0.024]. In addition, serum sodium, creatinine, blood urea nitrogen, and C-reactive protein levels were significantly higher in the SAE group (*P* < 0.05).

In terms of organ support, patients with SAE were more likely to receive vasopressor therapy [121 (56.28%) vs. 143 (44.41%), *P* = 0.007], mechanical ventilation [167 (77.67%) vs. 153 (47.52%), *P* < 0.001], and continuous renal replacement therapy [15 (6.98%) vs. 6 (1.86%), *P* = 0.003]. These findings suggest that SAE was associated with greater illness severity, respiratory dysfunction, renal impairment, systemic inflammation, and increased need for organ support among patients with sepsis.

### Feature selection

**Figure 2:**
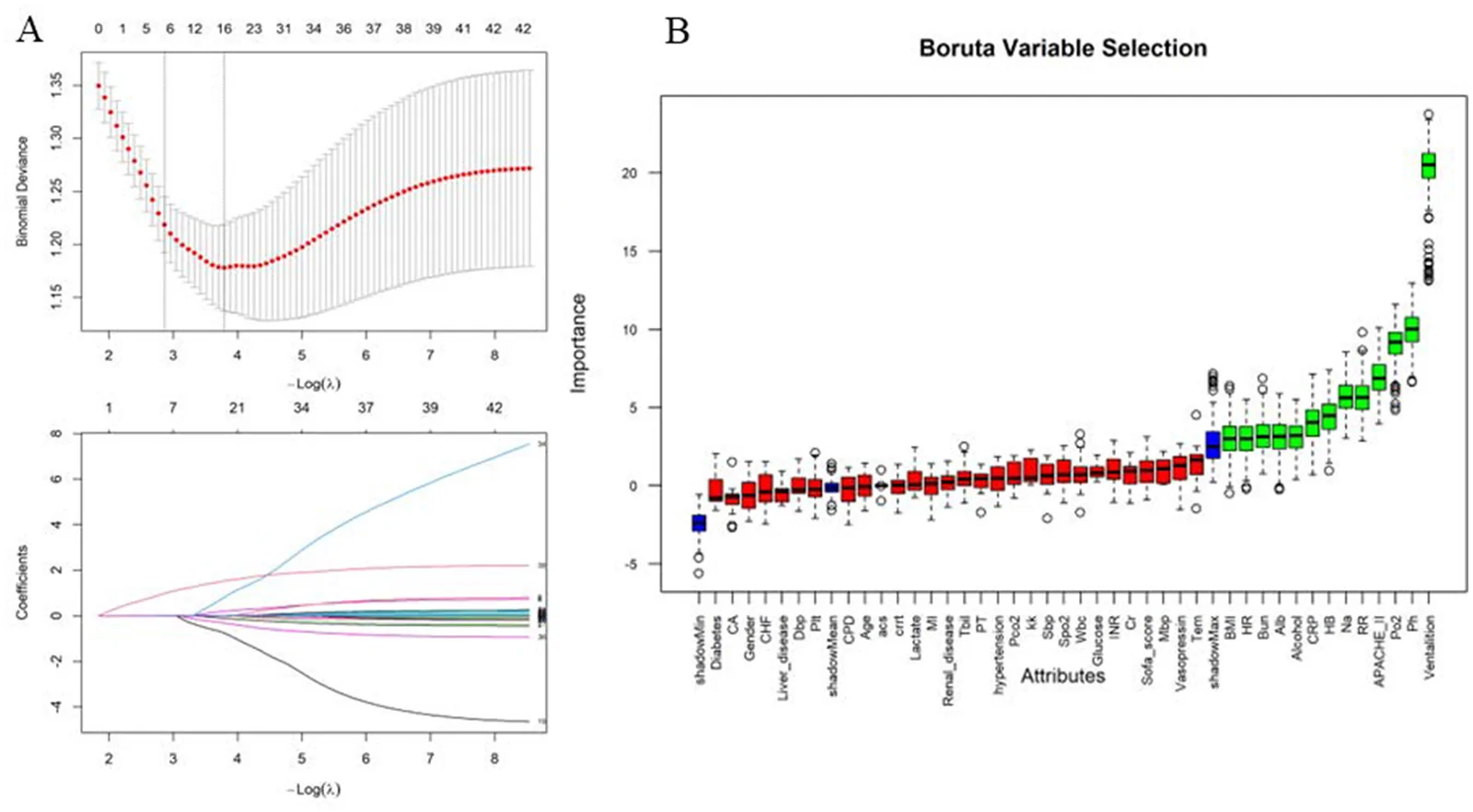
**(A)** Ten-fold cross-validation for the LASSO regression model. The upper plot shows the relationship between binomial deviance and log(λ), with the dotted vertical lines indicating the optimal λ values based on the minimum criterion and the one-standard-error criterion. The lower plot shows the coefficient profiles of candidate predictors as log(λ) changes, illustrating the gradual shrinkage of coefficients with increasing regularization. **(B)** Boruta variable selection results. Green boxes indicate confirmed important variables, red boxes indicate rejected variables, and blue boxes represent shadow attributes. Variables with higher importance scores were considered more relevant for predicting sepsis-associated encephalopathy.

The dataset was randomly split into a training set and an internal test set at a 7:3 ratio. Candidate predictors were prespecified based on clinical relevance and their availability within the first 24 h after ICU admission. To reduce model complexity and minimize the risk of overfitting, feature selection was performed on the training set using the Least Absolute Shrinkage and Selection Operator (LASSO) regression method and the Boruta method, and the common variables identified by both methods were selected (Figure 2). Nine predictors were retained: APACHE II score, respiratory rate, pH, PaO_2_, BUN, sodium, CRP, mechanical ventilation within 24 h, alcohol abuse. Feature selection was performed exclusively within the training set to prevent information leakage. The final prediction model was subsequently refitted using these predictors in the full training set and evaluated in the internal test set.

### Model performance comparisons in the internal test set

**Figure 3:**
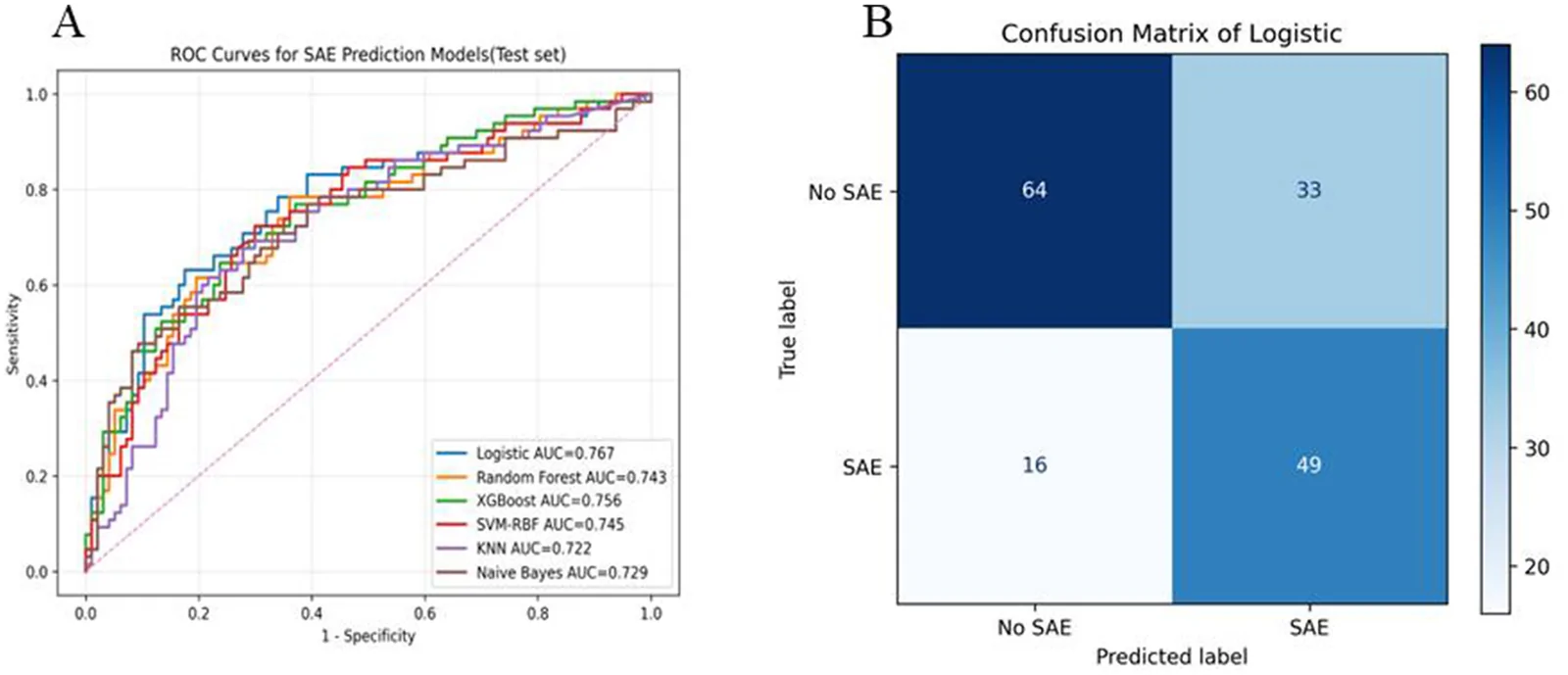
**(A)** Receiver operating characteristic (ROC) curves of the six candidate models in the internal test set. The logistic regression model achieved the highest AUC of 0.767, followed by XGBoost (AUC = 0.756), SVM-RBF (AUC = 0.745), random forest (AUC = 0.743), naive Bayes (AUC = 0.729), and KNN (AUC = 0.722). The dashed diagonal line represents a non-informative classifier. **(B)** Confusion matrix of the logistic regression model in the internal test set using a probability threshold of 0.5. Among patients without SAE, 64 were correctly classified as non-SAE and 33 were incorrectly classified as SAE. Among patients with SAE, 49 were correctly classified as SAE and 16 were incorrectly classified as non-SAE.

The predictive performance of six machine learning models is summarized in Table 2. In the internal test set, the logistic regression model achieved an AUC of 0.767 (95% CI: 0.688–0.845), PR-AUC of 0.699 (95% CI: 0.576–0.815), sensitivity of 0.754 (95% CI: 0.648–0.857), specificity of 0.660 (95% CI: 0.562–0.748), and Brier score of 0.201 (95% CI: 0.170–0.234). The calibration slope and calibration intercept were 0.843 and −0.484, respectively. Logistic regression showed the highest AUC in the internal test set, although its performance was comparable to that of XGBoost and other candidate models. Given the overlapping uncertainty in performance estimates, logistic regression was selected primarily because of its discrimination, calibration, and interpretability (Figure 3).

**Table 2:** Model performance comparisons.

Model	AUC	PR_AUC	Accuracy	Sensitivity	Specificity	Precision	F1
Logistic model	0.767	0.699	0.698	0.754	0.660	0.598	0.667
XGBoost	0.756	0.695	0.710	0.523	0.835	0.680	0.591
SVM-RBF	0.745	0.670	0.679	0.738	0.639	0.578	0.649
Random forest	0.743	0.659	0.673	0.646	0.691	0.583	0.613
Naive Bayes	0.729	0.676	0.673	0.769	0.608	0.568	0.654
KNN	0.722	0.598	0.716	0.615	0.784	0.656	0.635

AUC, Area under curve; SVM, support vector machine; KNN, K-nearest neighbor.

The XGBoost model showed a comparable AUC (0.756) and PR-AUC (0.695), and achieved the highest specificity among all models (0.835), but its sensitivity was relatively low (0.523), suggesting that it was more conservative in identifying SAE cases. The SVM-RBF model demonstrated moderate performance, with an AUC of 0.745, PR-AUC of 0.670, sensitivity of 0.738, and F1 score of 0.649. Random forest yielded an AUC of 0.743 and PR-AUC of 0.659, with relatively balanced sensitivity and specificity. Naive Bayes achieved the highest sensitivity (0.769), but its specificity was the lowest (0.608), indicating a tendency to classify more patients as SAE. KNN had the highest accuracy (0.716) and relatively high specificity (0.784), but its AUC (0.722) and PR-AUC (0.598) were lower than those of the other models.

### Sensitivity analysis

In the sensitivity analysis excluding mechanical ventilation, model performance decreased in the internal test set, with an AUC of 0.704, sensitivity of 0.462, and specificity of 0.845. Compared with the original model, this decrease in discrimination and sensitivity suggests that mechanical ventilation contributed substantially to risk classification. These results are presented in Supplementary Table S1.

In the primary analysis, the conventional APACHE II score was used as a candidate severity score. Because the conventional APACHE II score contains a GCS-derived neurological component, and GCS was included in the operational definition of SAE, we performed a sensitivity analysis to assess potential outcome-proxy contamination. The modified APACHE II score without GCS was calculated by subtracting the GCS-derived component from the conventional APACHE II score. Because the APACHE II neurological component is calculated as 15 minus the GCS score, the modified APACHE II score was calculated as: modified APACHE II = conventional APACHE II – (15 – GCS). In the sensitivity analysis, this modified APACHE II score was used in place of the conventional APACHE II score, and the model was refitted and internally validated. The model performance (Supplementary Figure S1) remained stable, with an AUC of 0.763, compared with 0.767 in the original model. This finding suggested that the predictive performance was not substantially driven by the neurological component of APACHE II.

### Calibration in the internal test set

**Figure 4:**
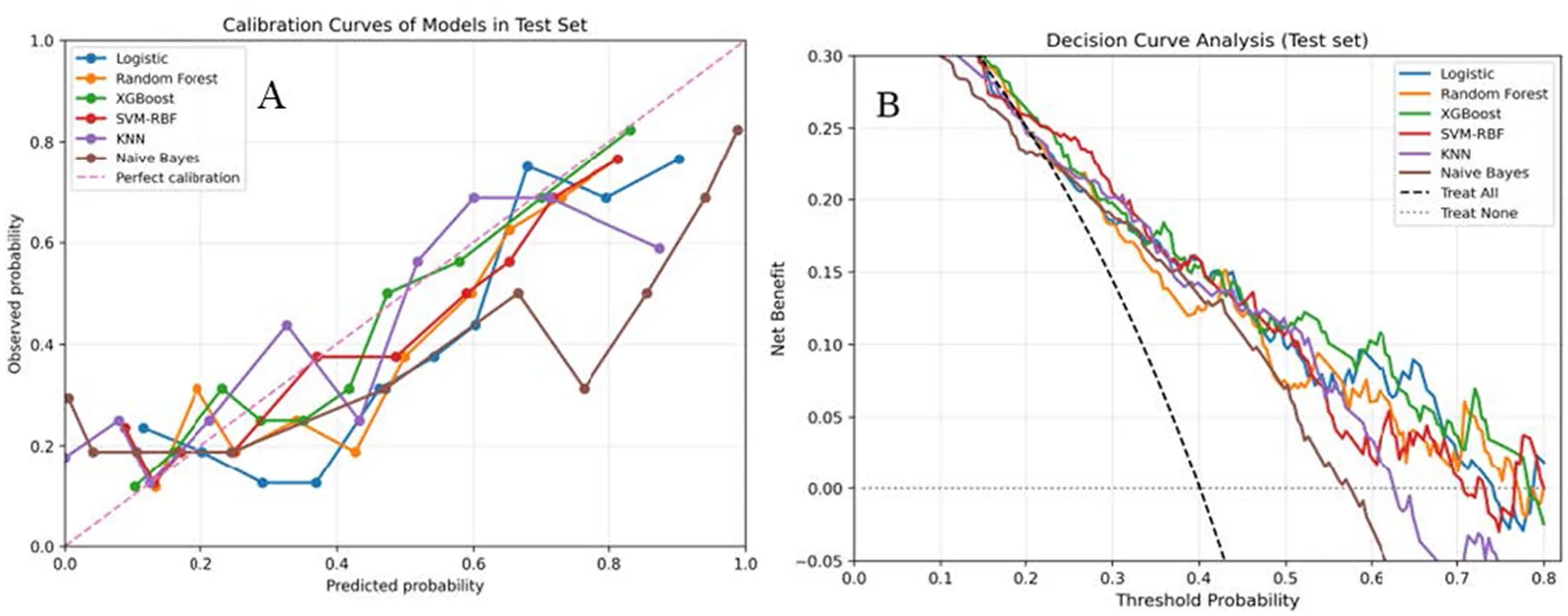
**(A)** Calibration curves of the six candidate models in the internal test set. The dashed diagonal line represents perfect agreement between predicted and observed SAE risk. Curves closer to the diagonal indicate better calibration. The logistic regression model showed generally acceptable agreement in the mid-to-high predicted-risk range, whereas deviations were observed in the low predicted-risk range, suggesting that predicted probabilities in low-risk patients should be interpreted cautiously. **(B)** Decision-curve analysis of the candidate models in the internal test set. The x-axis represents threshold probability, and the y-axis represents net benefit. The dashed black line represents the strategy of treating all patients as high risk, whereas the dotted gray line represents the strategy of treating no patients. The logistic regression model and other candidate models showed higher net benefit than the treat-none strategy across a broad range of threshold probabilities and exceeded the treat-all strategy over part of the clinically plausible threshold range.

The Brier score of the logistic regression model was 0.201, with a calibration slope of 0.843 and calibration intercept of −0.484 (Supplementary Table S2). The calibration curve (Figure 4) showed generally acceptable agreement between predicted and observed risks, although deviations were observed, particularly in the low predicted-risk range. Therefore, the estimated probabilities should be interpreted cautiously and require further validation.

### Potential net benefit assessed by decision curve analysis

Decision curve analysis (Figure 4) suggested that the logistic regression model provided a higher net benefit than the treat-all and treat-none strategies across threshold probabilities of approximately 0.15 to 0.74 in the internal test set. However, because this analysis was based on internal validation only, the potential net benefit of the model should be considered exploratory and requires confirmation in external and prospective cohorts.

### Model interpretability

**Figure 5:**
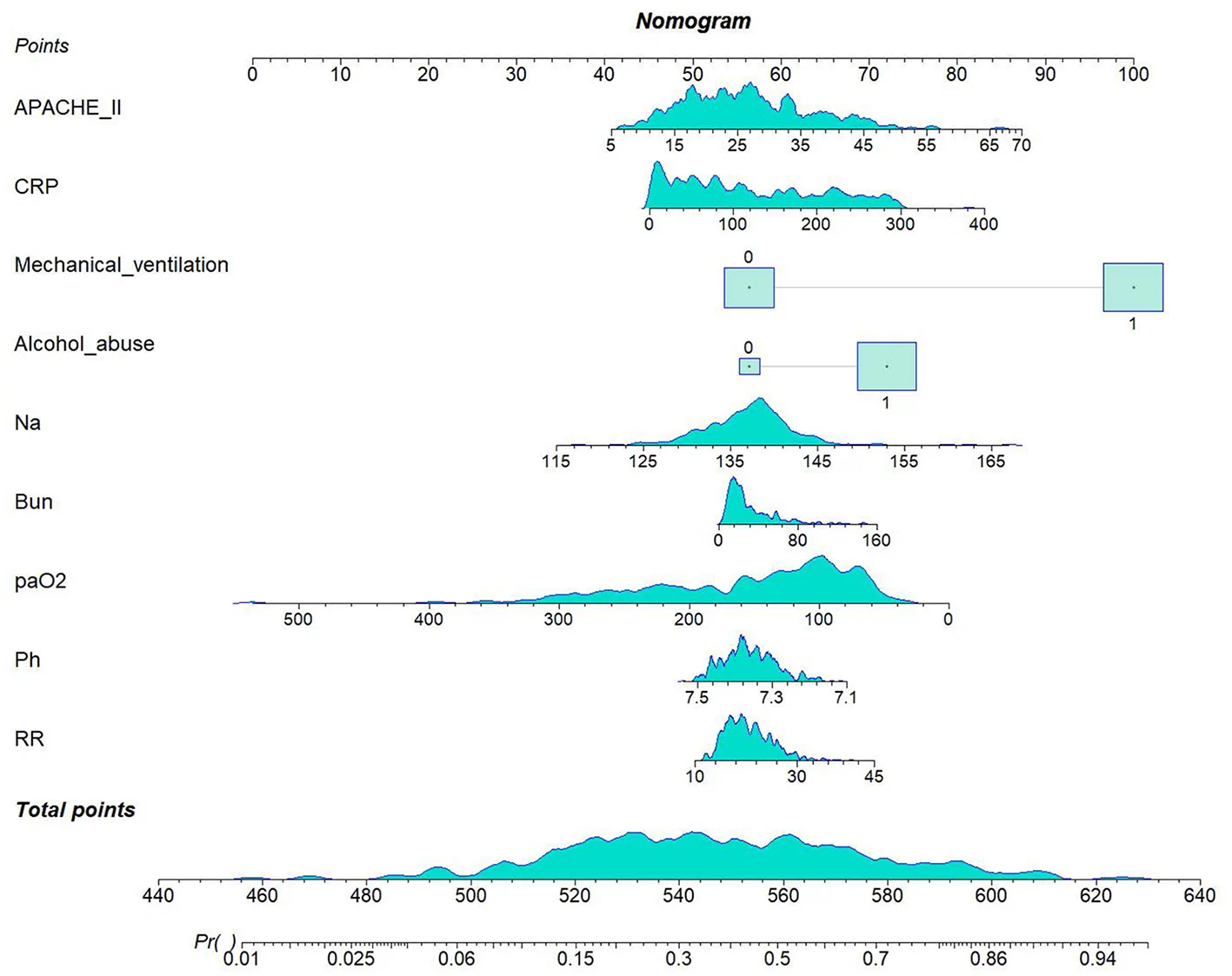
The nomogram was constructed based on the selected predictors, including APACHE II score, C-reactive protein (CRP), mechanical ventilation, alcohol use, serum sodium (Na), blood urea nitrogen (BUN), partial pressure of oxygen (PaO_2_), pH, and respiratory rate (RR). Each predictor is assigned a corresponding point value according to its contribution to the model. The total points are calculated by summing the points for all predictors and then mapped to the predicted probability of sepsis-associated encephalopathy. The blue density plots show the distribution of each predictor and the total score in the study population.

The results of the multivariate logistic regression are presented in Table 3. Based on the logistic regression results, we constructed a nomogram (Figure 5), which was developed to facilitate individualized prediction of SAE. The nomogram included APACHE II score, C-reactive protein, mechanical ventilation, alcohol use, serum sodium, blood urea nitrogen, PaO_2_, pH, and respiratory rate. By assigning points to each predictor and summing them into a total score, the nomogram estimated the individual probability of SAE. Higher total points were associated with a greater predicted risk of SAE, with predicted probabilities ranging from approximately 0.01 to 0.94, suggesting that the nomogram may serve as a preliminary risk stratification tool for SAE risk assessment.

**Table 3:** Multivariable logistic regression model.

Variables	β coefficient	OR	95% CI	*P*-value
APACHE II	0.032	1.033	1.005–1.060	0.017
RR	0.026	1.026	0.972–1.084	0.349
pH	−1.885	0.152	0.005–4.720	0.282
PaO_2_	−0.006	0.993	0.989–0.997	< 0.001
Bun	0.005	1.005	0.994–1.016	0.363
Na	0.044	1.045	0.998–1.095	0.063
CRP	0.004	1.004	1.001–1.007	0.004
Mechanical ventilation	1.950	7.022	3.949–12.486	< 0.001
Alcohol abuse	0.697	2.009	0.846–4.772	0.114

The model intercept was 4.484. ORs for continuous variables are expressed per one-unit increase. Mechanical ventilation and alcohol abuse were coded as yes vs. no; RR, respiratory rate; CRP, C-reactive protein; APACHE II, Acute Physiology and Chronic Health Evaluation II.

## Discussion

In this study, we developed and internally validated a 24-hour landmark model to predict new-onset SAE after the first ICU day among patients with sepsis who survived, remained in the ICU, and were SAE-free during the first 24 h after ICU admission. Therefore, the model should not be interpreted as an ICU-admission model or as a tool for immediate prediction at ICU entry.

SAE is a common manifestation of acute brain dysfunction in sepsis and is associated with increased mortality, prolonged ICU stay, and long-term cognitive impairment (15). Its pathogenesis is multifactorial, involving systemic inflammation, endothelial injury, blood-brain barrier disruption, impaired cerebral perfusion, mitochondrial dysfunction, neurotransmitter imbalance, and metabolic derangements (16, 17). Because no single diagnostic biomarker is currently available, early identification depends on integrating clinical, physiological, and laboratory information. Although logistic regression achieved the highest AUC in the internal test set, its advantage over XGBoost and other algorithms was modest. Therefore, we do not interpret logistic regression as definitively superior. Instead, it was selected as the final model because it provided comparable discrimination, acceptable calibration, and better interpretability, which is important for clinical risk stratification. The decision-curve analysis suggested potential net benefit within a threshold probability range of approximately 0.15 to 0.74. Nevertheless, this finding should be interpreted cautiously because it was derived from a single-center internally validated cohort. The model should therefore be considered a preliminary risk stratification tool rather than a bedside-ready clinical decision aid.

Among the variables included in the final model, APACHE II score reflects overall illness severity and has been widely used for prognosis assessment in critically ill patients (18). In our model, higher APACHE II score contributed to increased predicted SAE risk, indicating that patients with more severe systemic illness are more vulnerable to acute brain dysfunction. This finding is biologically plausible, as severe sepsis is often accompanied by hemodynamic instability, tissue hypoperfusion, inflammatory activation, and multi-organ dysfunction, all of which may contribute to cerebral injury (19). A potential concern is that APACHE II includes a neurological component derived from GCS, while GCS was also used in the operational definition of SAE. To reduce the risk of label leakage, we recalculated APACHE II after removing the GCS component and repeated the model analysis. The AUC remained essentially unchanged, decreasing only from 0.767 to 0.763. This supports the robustness of the model and suggests that its performance was not primarily attributable to neurological information embedded in APACHE II. Nevertheless, future studies using prospectively adjudicated SAE outcomes and severity scores independent of neurological assessment are warranted. Similarly, CRP was included as an inflammatory marker. Elevated CRP may reflect a stronger systemic inflammatory response, which can promote endothelial activation, blood-brain barrier dysfunction, and neuroinflammation, thereby increasing the likelihood of SAE (20). Respiratory rate, pH, PaO_2_, and mechanical ventilation within 24 h suggest that respiratory dysfunction plays an important role in SAE risk. Tachypnea is a component of sepsis-related bedside risk assessment and reflects early physiological stress in sepsis (1). Hypoxemia may directly impair cerebral oxygen delivery, while acid-base disturbances can alter cerebral blood flow, neuronal excitability, and consciousness (6, 21). Mechanical ventilation should be interpreted cautiously. Although it was one of the strongest predictors in the original model, it may not represent a direct causal factor for SAE. Instead, it may serve as a composite marker of respiratory failure, greater illness severity, sedative and analgesic exposure, reduced arousal, and the practical difficulty of neurological assessment in ventilated patients (22, 23). In the sensitivity analysis excluding mechanical ventilation, model performance decreased from an AUC of 0.767 to 0.704, and sensitivity decreased from 0.754 to 0.462. This indicates that mechanical ventilation carries important predictive information but also highlights the possibility of assessment bias and residual confounding. Therefore, this variable should be used for risk stratification rather than causal interpretation. Thus, early respiratory deterioration should alert clinicians to the possibility of SAE and prompt structured neurological monitoring. Renal dysfunction and metabolic abnormalities were also represented in the model by BUN and sodium. Elevated BUN may reflect acute kidney injury, catabolic stress, dehydration, or impaired clearance of neurotoxic metabolites. Prior multicenter evidence has identified acute renal failure and metabolic disturbances as potentially modifiable contributors to SAE (5). Sodium disturbance, particularly hypernatremia, may promote neuronal dehydration, osmotic stress, and altered mental status (6, 21). These findings support the concept that SAE is not only an inflammatory brain injury but also a systemic metabolic encephalopathy in which reversible factors may be clinically actionable (5, 6, 21). Alcohol use was also retained in the model. Chronic alcohol exposure may influence SAE risk through several mechanisms, including baseline neurological vulnerability, nutritional deficiency, immune dysregulation, hepatic dysfunction, withdrawal syndrome, and increased susceptibility to infection or organ dysfunction (24). Although alcohol use may be influenced by documentation bias and heterogeneity in exposure definition, its inclusion suggests that premorbid factors may also contribute to neurological outcomes in patients with sepsis.

An important strength of the present model is its interpretability. Although complex machine learning algorithms are often expected to provide superior predictive performance, logistic regression showed the highest AUC in the internal test set in our study. This may be related to the moderate sample size, the use of clinically meaningful structured variables, and the relatively stable linear associations between selected predictors and SAE risk. Compared with more complex black-box models, logistic regression provides transparent coefficients and can be easily translated into a nomogram (25), making it more interpretable for preliminary clinical risk stratification. The nomogram further allows clinicians to estimate individual SAE risk using routinely collected variables, which may support enhanced neurological monitoring after the 24-hour landmark time point, optimization of oxygenation and acid-base status, correction of metabolic disturbances, and targeted prevention strategies. It is noteworthy that, a high predicted risk should not be interpreted as a definitive diagnosis of SAE, but rather as a trigger for enhanced neurological surveillance, optimization of oxygenation and ventilation, correction of electrolyte and acid-base abnormalities, renal function management, alcohol withdrawal prevention, sedation minimization, and implementation of prevention bundles (5, 23).

This study has several limitations. First, the retrospective single-center design may have introduced selection bias and information bias. In addition, because patients who died, were discharged, or developed SAE within the first 24 h after ICU admission were excluded, the model should be interpreted as a 24-hour landmark prediction model. Therefore, it applies only to patients who survived, remained in the ICU, and were free of SAE during the first ICU day. This design may introduce survivorship bias and limit generalizability to patients with very early SAE or rapidly fatal sepsis. Second, sedation-related confounding remains an important limitation. The operational definition of SAE was based on GCS and CAM-ICU assessments, both of which may be affected by sedative and analgesic exposure, sedation depth, mechanical ventilation, and fluctuating arousal. Although patients with direct central nervous system infection, structural brain disease, severe hypoglycemia, hepatic encephalopathy, uremic encephalopathy, and drug intoxication were excluded, GCS/CAM-ICU-defined SAE could not be completely disentangled from sedation-related impaired arousal in this retrospective dataset. In particular, mechanical ventilation may reflect greater illness severity and respiratory failure, but it is also closely related to sedative and analgesic exposure, which can directly alter consciousness level and influence GCS and CAM-ICU assessments. Because detailed information on sedative and analgesic agents, cumulative sedative dose, Richmond Agitation-Sedation Scale scores, and sedation depth was not consistently available, residual confounding and outcome misclassification related to sedation and mechanical ventilation could not be fully excluded. Third, although the model demonstrated acceptable discrimination and calibration in the internal test set, the validation strategy was limited to a single-center cohort with a random train-test split. Therefore, the model should be considered a preliminary internally validated risk stratification tool rather than a bedside-ready clinical decision aid. External validation in independent multicenter cohorts, preferably with temporally distinct data, and prospective impact studies are required before clinical implementation. Fourth, some potentially relevant predictors, such as sedative exposure, electroencephalography findings, neuroimaging, infection source, inflammatory cytokines, and dynamic changes in physiological and laboratory variables, were not included or were not consistently available. Finally, the model was developed using variables collected during the first 24 h after ICU admission. Future studies should explore whether dynamic prediction models incorporating time-series ICU data can further improve SAE risk assessment.

## Conclusion

In conclusion, we developed and internally validated a 24-hour landmark model for predicting new-onset SAE after the first ICU day in patients with sepsis. The model showed moderate discrimination and exploratory potential net benefit in the internal test set. However, given the single-center retrospective design and lack of external validation, it should be considered a preliminary risk stratification tool. Independent external validation in multicenter and temporally distinct cohorts is required before clinical implementation.

## Data Availability

The data analyzed in this study is subject to the following licenses/restrictions. The raw data supporting the conclusions of this article will be made available by the authors, without undue reservation. Requests to access these datasets should be directed to Ziming Jiang, yydsjzm@163.com.

## References

[1] Singer M Deutschman CS Seymour CW Shankar-Hari M Annane D Bauer M . The third international consensus definitions for sepsis and septic shock (Sepsis-3). JAMA. (2016) 315:801–10. doi: 10.1001/jama.2016.0287 26903338 PMC4968574

[2] Rudd KE Johnson SC Agesa KM Shackelford KA Tsoi D Kievlan DR . Global, regional, and national sepsis incidence and mortality, 1990–2017: analysis for the Global Burden of Disease Study. Lancet. (2020) 395:200–11. doi: 10.1016/S0140-6736(19)32989-7 31954465 PMC6970225

[3] Evans L Rhodes A Alhazzani W Antonelli M Coopersmith CM French C . Surviving sepsis campaign: international guidelines for management of sepsis and septic shock 2021. Intensive Care Med. (2021) 47:1181–247. doi: 10.1007/s00134-021-06506-y 34599691 PMC8486643

[4] Chung HY Wickel J Brunkhorst FM Geis C. Sepsis-associated encephalopathy: from delirium to dementia? J Clin Med. (2020) 9:703. doi: 10.3390/jcm9030703 32150970 PMC7141293

[5] Sonneville R Benghanem S Jeantin L de Montmollin E Doman M Gaudemer A . The spectrum of sepsis-associated encephalopathy: a clinical perspective. Crit Care. (2023) 27:386. doi: 10.1186/s13054-023-04655-8 37798769 PMC10552444

[6] Gofton TE Young GB. Sepsis-associated encephalopathy. Nat Rev Neurol. (2012) 8:557–66. doi: 10.1038/nrneurol.2012.183 22986430

[7] Ren C Yao RQ Zhang H Feng YW Yao YM. Sepsis-associated encephalopathy: a vicious cycle of immunosuppression. J Neuroinflammation. (2020) 17:14. doi: 10.1186/s12974-020-1701-3 31924221 PMC6953314

[8] Iwashyna TJ Ely EW Smith DM Langa KM. Long-term cognitive impairment and functional disability among survivors of severe sepsis. JAMA. (2010) 304:1787–94. doi: 10.1001/jama.2010.1553 20978258 PMC3345288

[9] Catarina AV Branchini G Bettoni L De Oliveira JR Nunes FB. Sepsis-associated encephalopathy: from pathophysiology to progress in experimental studies. Mol Neurobiol. (2021) 58:2770–9. doi: 10.1007/s12035-021-02303-2 33495934

[10] Lu X Kang H Zhou D Li Q. Prediction and risk assessment of sepsis-associated encephalopathy in ICU based on interpretable machine learning. Sci Rep. (2022) 12:22621. doi: 10.1038/s41598-022-27134-6 36587113 PMC9805434

[11] Peng L Peng C Yang F Wang J Zuo W Cheng C . Machine learning approach for the prediction of 30-day mortality in patients with sepsis-associated encephalopathy. BMC Med Res Methodol. (2022) 22:183. doi: 10.1186/s12874-022-01664-z 35787248 PMC9252033

[12] Collins GS Dhiman P Andaur Navarro CL Ma J Hooft L Reitsma JB . Protocol for development of a reporting guideline (TRIPOD-AI) and risk of bias tool (PROBAST-AI) for diagnostic and prognostic prediction model studies based on artificial intelligence. BMJ Open. (2021) 11:e048008. doi: 10.1136/bmjopen-2020-048008 PMC8273461 34244270

[13] Collins GS Reitsma JB Altman DG Moons KG. Transparent reporting of a multivariable prediction model for individual prognosis or diagnosis (TRIPOD): the TRIPOD statement. BMJ. (2015) 350:g7594. doi: 10.1136/bmj.g7594 25569120

[14] Zhang Z. Multiple imputation with multivariate imputation by chained equation (MICE) package. Ann Transl Med. (2016) 4:30. doi: 10.3978/j.issn.2305-5839.2015.12.63 26889483 PMC4731595

[15] Gao Q Hernandes MS. Sepsis-associated encephalopathy and blood-brain barrier dysfunction. Inflammation. (2021) 44:2143–50. doi: 10.1007/s10753-021-01501-3 34291398 PMC11044530

[16] Abbott NJ Rönnbäck L Hansson E. Astrocyte-endothelial interactions at the blood-brain barrier. Nat Rev Neurosci. (2006) 7:41–53. doi: 10.1038/nrn1824 16371949

[17] Daneman R Prat A. The blood-brain barrier. Cold Spring Harb Perspect Biol. (2015) 7:a020412. doi: 10.1101/cshperspect.a020412 25561720 PMC4292164

[18] Chen J Shi X Diao M Jin G Zhu Y Hu W . A retrospective study of sepsis-associated encephalopathy: epidemiology, clinical features and adverse outcomes. BMC Emerg Med. (2020) 20:77. doi: 10.1186/s12873-020-00374-3 33023479 PMC7539509

[19] Zhao Q Xiao J Liu X Liu H. The nomogram to predict the occurrence of sepsis-associated encephalopathy in elderly patients in the intensive care units: a retrospective cohort study. Front Neurol. (2023) 14:1084868. doi: 10.3389/fneur.2023.1084868 36816550 PMC9932587

[20] Yu D Liu J Song X Ao Y Li X Han Y. Analysis of the inflammatory storm response and heparin binding protein levels for the diagnosis and prognosis of sepsis-associated encephalopathy. Eur J Med Res. (2025) 30:116. doi: 10.1186/s40001-025-02369-x 39966958 PMC11834667

[21] Chaudhry N Duggal AK. Sepsis associated encephalopathy. Adv Med. (2014) 2014:762320. doi: 10.1155/2014/762320 26556425 PMC4590973

[22] Aarskog NR Bjørsrud HOL Holten AR Watne LO Neerland BE Rostrup M. Ventilatory response and delirium risk in hospitalised patients with acute hypoxia due to COVID-19. Sci Rep. (2025) 15:28309. doi: 10.1038/s41598-025-13016-0 40754538 PMC12319066

[23] Ely EW Inouye SK Bernard GR Gordon S Francis J May L . Delirium in mechanically ventilated patients: validity and reliability of the confusion assessment method for the intensive care unit (CAM-ICU). JAMA. (2001) 286:2703–10. doi: 10.1001/jama.286.21.2703 11730446

[24] Stewart D Kinsella J McPeake J Quasim T Puxty A. The influence of alcohol abuse on agitation, delirium and sedative requirements of patients admitted to a general intensive care unit. J Intensive Care Soc. (2019) 20:208–15. doi: 10.1177/1751143718787748 31447913 PMC6693117

[25] Grimes DA. The nomogram epidemic: resurgence of a medical relic. Ann Intern Med. (2008) 149:273–5. doi: 10.7326/0003-4819-149-4-200808190-00010 18711159

