# Supplementary material for: A 24-hour landmark machine learning model for predicting new-onset sepsis-associated encephalopathy
Source: Front Med (Lausanne). 2026 Aug 11;13:1904524. doi: 10.3389/fmed.2026.1904524 (PMC13503553; doi:10.3389/fmed.2026.1904524)
Supplement: Supplementary file 1 [file Supplementary_file_1.docx]

| **Supplementary Table S1.** Sensitivity analyses of model performance in the internal test set | | | | | | |
| --- | --- | --- | --- | --- | --- | --- |
| Model | AUC | Accuracy | Sensitivity | Specificity | Precision | F1 |
| Original model | 0.767 | 0.698 | 0.754 | 0.660 | 0.598 | 0.667 |
| Model without mechanical ventilation | 0.704 | 0.691 | 0.462 | 0.845 | 0.667 | 0.545 |
| Model without GCS in APACHE II | 0.763 | 0.728 | 0.600 | 0.814 | 0.684 | 0.639 |

AUC, area under the receiver operating characteristic curve; GCS, Glasgow Coma Scale; APACHE II, Acute Physiology and Chronic Health Evaluation II.
