# Supplementary material for: A 24-hour landmark machine learning model for predicting new-onset sepsis-associated encephalopathy
Source: Front Med (Lausanne). 2026 Aug 11;13:1904524. doi: 10.3389/fmed.2026.1904524 (PMC13503553; doi:10.3389/fmed.2026.1904524)
Supplement: Supplementary file 2 [file Supplementary_file_2.docx]

| **Supplementary Table S2.** Performance of candidate models in the internal test set with 95% confidence intervals | | | | | | | | | | |
| --- | --- | --- | --- | --- | --- | --- | --- | --- | --- | --- |
| Model | AUC | PR_AUC | Accuracy | Sensitivity | Specificity | Precision | F1 | Brier | Calibration slope | Calibration intercept |
| Logistic | 0.767 (0.688-0.845) | 0.699 (0.576-0.815) | 0.698 (0.623-0.765) | 0.754 (0.648-0.857) | 0.660 (0.562-0.748) | 0.598 (0.494-0.704) | 0.667 (0.575-0.752) | 0.201 (0.170-0.234) | 0.843 (0.530-1.298) | -0.484 (-0.874--0.140) |
| XGBoost | 0.756 (0.682-0.835) | 0.695 (0.573-0.810) | 0.710 (0.642-0.784) | 0.523 (0.397-0.643) | 0.835 (0.759-0.908) | 0.680 (0.544-0.811) | 0.591 (0.481-0.694) | 0.193 (0.161-0.223) | 0.936 (0.609-1.437) | -0.087 (-0.444-0.287) |
| SVM-RBF | 0.745 (0.670-0.827) | 0.670 (0.558-0.788) | 0.704 (0.636-0.778) | 0.692 (0.582-0.806) | 0.711 (0.619-0.804) | 0.616 (0.507-0.725) | 0.652 (0.557-0.743) | 0.201 (0.165-0.233) | 0.767 (0.500-1.165) | -0.176 (-0.545-0.174) |
| Random Forest | 0.743 (0.668-0.821) | 0.659 (0.533-0.785) | 0.673 (0.605-0.747) | 0.646 (0.531-0.761) | 0.691 (0.602-0.782) | 0.583 (0.471-0.689) | 0.613 (0.512-0.707) | 0.205 (0.174-0.234) | 0.892 (0.567-1.355) | -0.319 (-0.685-0.019) |
| Naive Bayes | 0.729 (0.642-0.811) | 0.676 (0.561-0.792) | 0.673 (0.599-0.747) | 0.769 (0.667-0.869) | 0.608 (0.510-0.707) | 0.568 (0.462-0.670) | 0.654 (0.556-0.741) | 0.249 (0.199-0.303) | 0.216 (0.084-0.446) | -0.407 (-0.790--0.081) |
| KNN | 0.722 (0.645-0.806) | 0.598 (0.490-0.732) | 0.716 (0.648-0.790) | 0.615 (0.507-0.735) | 0.784 (0.706-0.865) | 0.656 (0.533-0.780) | 0.635 (0.533-0.732) | 0.217 (0.176-0.256) | 0.135 (0.051-0.468) | -0.230 (-0.573-0.130) |

Data are presented as estimate (95% confidence interval). AUC, area under the receiver operating characteristic curve; PR-AUC, precision-recall area under the curve; F1, F1 score; Brier, Brier score.
