# Supplementary material for: A 24-hour landmark machine learning model for predicting new-onset sepsis-associated encephalopathy
Source: Front Med (Lausanne). 2026 Aug 11;13:1904524. doi: 10.3389/fmed.2026.1904524 (PMC13503553; doi:10.3389/fmed.2026.1904524)
Supplement: Supplementary file 3 [file Supplementary_file_3.docx]

| **Table S3**. Multivariable Logistic Regression Model | | | | | |
| --- | --- | --- | --- | --- | --- |
| Variables |  | β coefficient | OR | 95% CI | *P*-value |
| APACHE II (per 10-point increase) | | 0.32 | 1.38 | 1.06-1.80 | 0.02 |
| RR (per 5 breaths/min increase) | | 0.13 | 1.14 | 0.87-1.50 | 0.35 |
| pH | | -1.89 | 0.15 | 0.01-4.72 | 0.28 |
| PaO_2_ (per 10 mmHg increase) | | -0.07 | 0.94 | 0.90-0.97 | 0.001 |
| Bun (per 10-unit increase) | | 0.05 | 1.05 | 0.94-1.17 | 0.36 |
| Na (per 10 mmol/L increase) | | 0.44 | 1.56 | 0.98-2.50 | 0.06 |
| CRP (per 10-unit increase) | | 0.04 | 1.04 | 1.01-1.08 | 0.004 |
| Mechanical ventilation | | 1.95 | 7.02 | 4.01-12.71 | <0.001 |
| Alcohol abuse | | -0.70 | 0.50 | 0.20-1.15 | 0.11 |

The model intercept was 5.12. Mechanical ventilation and alcohol abuse were coded as yes versus no; RR: Respiratory rate; CRP: C-reactive protein; APACHE II: Acute Physiology and Chronic Health Evaluation II;
